# Physicochemical Composition and Apparent Degree of Polymerization of Fructans in Five Wild Agave Varieties: Potential Industrial Use

**DOI:** 10.3390/foods8090404

**Published:** 2019-09-12

**Authors:** Pamela I. Aldrete-Herrera, Mercedes G. López, Luis Medina-Torres, Juan A. Ragazzo-Sánchez, Montserrat Calderón-Santoyo, Marisela González-Ávila, Rosa I. Ortiz-Basurto

**Affiliations:** 1 Laboratorio Integral de Investigación en Alimentos, Tecnológico Nacional de México - Instituto Tecnológico de Tepic, Av. Tecnológico 2595 Fracc. Lagos del Country, 63175 Tepic, Nayarit, Mexico; paisaldereteherrera@ittepic.edu.mx (P.I.A.-H.); jragazzo@ittepic.edu.mx (J.A.R.-S.);; 2 Centro de Investigación y de Estudios Avanzados del IPN. Km. 9.6 Libramiento Norte Carretera Irapuato León, 36821 Irapuato, Guanajuato, Mexico; mercedes.lopez@cinvestav.mx; 3 Facultad de Química de la Universidad Nacional Autónoma de México, Circuito Exterior S/N, Coyoacán, Cd. Universitaria, 04510 México city, Mexico; luismt@unam.mx; 4 Centro de Investigación y Asistencia en Tecnología y diseño del Estado de Jalisco, Av. Normalistas 800, Colinas de La Normal, 44270 Guadalajara, Jalisco, Mexico; mgavila@ciatej.mx

**Keywords:** wild agave plants, agave fructans, apparent degree of polymerization, agave age

## Abstract

In this study, we characterize fructan extracts from five wild agave varieties at three ages to identify their potential use in the food industry. Physicochemical parameters (solids soluble total and pH), sugar content and fructan distribution profiles by high-performance anion-exchange chromatography (HPAEC) were evaluated. We found that the ages and variety influenced the carbohydrate content and also fructan dispersion. Two- to four-year-old plants exhibited the highest concentrations of free sugars and fructans, with a low apparent degree of polymerization (DP_a_) of ≤9 monomers, which highlights their potential use as prebiotics. Conversely, 10- to 12-year-old plants presented a low concentration of free sugars and fructans with a maximum DP_a_ of 70 monomers, which can be used to obtain fractions with high, intermediate and low DP_a_. These fractions have a potential use in the food industry as prebiotic, soluble fibers, stabilizers and sweeteners, among others. The agave varieties *Agave* spp., *Agave salmiana*, and *Agave atrovirens* showed mainly fructooligosaccharides (FOSs). Due to the presence of these low molecular carbohydrates, prebiotics, fermented products and/or syrups could be obtained. *A. salmiana* spp. crassipina and *Agave tequilana* variety cenizo presented DP_a_ ≤50 and DP_a_ ≤70, respectively, which could be useful in the production of fructan fractions of different DP_a._ These fractions might be used as functional ingredients in the manufacture of a wide range of food products.

## 1. Introduction 

Mexico has a large diversity of agave species distributed mainly in arid zones, which have been used for the development of alcoholic beverages, syrup and agave fructans. Agave fructans are fructose polymers derived from sucrose with β (2→1) and/or β (2→6) linkages that can contain terminal or intermediate glucose. They act as reserve carbohydrates in 15% of superior plants [1]. 

Due to their nutritional and technological characteristics, the use of agave fructans as healthy additives in foods continue gaining interest in the food industry because of their prebiotic benefits as soluble dietary fiber, as well as their technological functions as stabilizers and sweeteners, among other applications [2,3,4,5,6,7,8,9].

In addition, it has been demonstrated that agave fructans increase the concentration of *Lactobacill* in the large intestine [10]. These health effects might be related to the dispersion of the degree of polymerization and to the complexity of the linkage type [11,12]. Dispersion, concentration and linkage type are influenced by the agave species’ diversity, and climatic factors such as hydric stress, low temperatures and/or nitrogen deficiency; these factors can quickly change the production of fructans in the plant. These factors also induce the activity of fructosyltransferase as a self-preservation response [13,14].

Some studies have demonstrated that the agave fructans from diverse species have the largest number of complex isomers with different degree of polymerization [10,15]. Furthermore, agave species possess a mixture of different types of branched and linear fructans [1]. Independently of the large diversity of agave species, most studies have been focused on the *Agave tequilana* Weber Blue variety with Denomination of Origin Tequila (DOT) for the production of tequila and agave native fructans. Nowadays, an increasing demand for agave fructans exists due to the prebiotic and technological effects demonstrated by native agave fructans and enriched DP fractions [16].

Taking this into account, the objective of this study was to characterize the fructan extracts from five wild agave varieties at three ages, and to identify their potential use as raw materials in the production of native agave fructans, enriched DP fractions, agave syrup and/or fermented beverages. 

## 2. Materials and Methods

### 2.1. Materials

Five wild agave varieties at three different ages (2–4 years-old, 6–8 years-old and 10–12 years old) were collected according to the methodology proposed by Arrizon, Morel, Gschaedler and Monsan (2010) [17], with some modifications. The varieties were *A.* spp., *A. atrovirens* and *A. mapisaga* spp. crassipina from Perote, Veracruz (19°33′43″N97°14′31″O), *A. tequilana* cenizo variety from Jalisco (20°34′00″N103°40′35″O) and *A. salmiana* chino variety from San Felipe, Guanajuato (21°28′51″N 101°12′49″O). We collected a portion of the stems and leaf bases from each plant and stored samples in plastic bags at −20 °C.

### 2.2. Extraction

The extraction of agave fructans from the stems and leaves of each plant was carried out with ethanol 80% (*v*/*v*) at 60 °C under constant agitation for 30 min. Thereafter, we performed two washes with distilled water for 15 min at 60 °C [18].

### 2.3. Physicochemical Properties

The determination of pH and total soluble solids (TSS) were performed according to the Association of Official Analytical Chemist (AOAC-1990). Direct reducing sugars (DRSs) and agave fructans were determined using the fructans HK enzymatic kit (MEGAZYME Int. Ireland, Ireland) with a fructose standard as reference; samples were analyzed at a wavelength of 340 nm in a spectrophotometer (Varian Cary 50 Bio, UV-Vis, Walnut Creek, CA, USA). All measures were carried out in triplicate.

### 2.4. Mid-Infrared Spectroscopy (Mid-Ir) and Principal Component Analysis (Pca)

Fructan extracts from each variety were prepared using 2 mg·mL^−1^ and filtered through a 0.45-μm membrane. The analyses were performed with Spectrum Quant software (Perkin Elmer, Beaconsfield, Buckinghamshire, UK) at a wavelength from 4000 to 650 cm^−1^ in transmittance mode (%T). The readings were carried out in triplicate for each sample. PCA clusters were done using only the wavelength range specific for carbohydrates (1300–900 cm^−1^) and analyzed with STATISTICA v.12.0 software (Stat Soft. Inc. 1984–2014, Tulsa, OK, USA). Due to the difficulty of knowing the exact plant age and therefore the possible change on carbohydrate concentration, a PCA analysis was made for the plant replicates at different ages.

### 2.5. Fructas Distribution Profiles

#### High-Performance Anion-Exchange Chromatography (HPAEC)

The distribution profiles of fructans were carried out in a liquid ion-exchange chromatograph DIONEX ICS-5000 (Thermoscientific, Waltham, MA, USA), using a Carbopac PA-100 (40 × 250 mm) column with a precolumn (40 × 25 mm) with a gradient of 100 mM NaOH and 600 mM CH_3_COONa for 90 minutes at 35 °C. Chicory inulin (SIGMA ALDRICH I-2255, St. Louis, MO, USA) was used as a standard. Additionally, standards of glucose, fructose, sucrose, kestose and nystose (SIGMA ALDRICH, St. Louis, MO, USA) were included to complete the distribution profile. Prior to their injection, samples were diluted in mili-Q water (2 mg·mL^−1^) and filtered through a 0.45 µm (Millipore^®^, Burlington, MA, USA) nylon membrane; the obtained results were expressed in nC versus time.

### 2.6. Statistical Analysis

The analyses were performed using an analysis of variance (ANOVA). The least significant difference at *p* < 0.05 was calculated using a Fisher test using the statistical software STATISTICA 12.0 (StatSoft. Inc. 1984–2014, Tulsa, OK, USA). All the experiments were performed in triplicate and the data were expressed with the means and standard deviations (SD).

## 3. Results and Discussion

### 3.1. Physicochemical Properties 

The collection of five wild agave varieties was carried out in three groups using the approximate age of the plants for their classification (Table 1). The length from top to top of the leaves was considered to have a greater homogeneity among the samples.

Table 1 also shows the sugar contents of the five wild agave varieties; a statistically significant difference was observed (*p* = 0.005049) with an increase in fructans concentration and leaf to leaf length based on plant age. The agave plants between 2–4 years of age showed a higher concentration of direct reducing sugar (DRS) (maximum of 26.4 g of sugar/100 g of dry agave) compared to the adult plants. In 6- to 8-year-old plants, the presence of DRS decreased, presenting a maximum concentration of 18.55 g of sugars/100 g of agave. Finally, in the 10- to 12-year-old plants, a decrease in DRS was again observed, and an increase up to 89.7% in fructans was found. The differences of carbohydrate concentrations show that in the first two growth stages, a greater accumulation of DRS existed; in the early stage (2–4 years old) the plants used carbohydrates in their simplest form (glucose, fructose and sucrose) as energy reserve for their growth. At this stage, there was also greater activity of the fructan exohydrolase enzymes (FEH), which are responsible for fructan hydrolysis and also sucrose invertase, producing the needed glucose and fructose forms readily accessible to the plant [1,19,20]. This behavior was observed in all studied agave varieties at this age. On the other hand, the carbohydrate concentration and fructan dispersion increased as a function of plant age, mainly due to the activity of fructosyltransferases; these enzymes regulate their activity depending on the growing stage of the plant and are in charge of the polymerizing and branching of the fructan chains. Van den Ende and Van Laere (1996) [21] reported that the activity of fructosyltransferases varies in each species, and that their activities depend on the accumulation of substrate in the plant and the surrounding climatic conditions. In the case of *A. tequilana* Weber Blue variety, an action model of enzymes of each age has been proposed in which the adult stages present a high activity of sucrose: fructan 6-fructosyltransferase (6-SFT) and fructan: fructan 1-fructosyltransferase (1-FFT) enzymes before the flowering that permits the formation and accumulation of fructan chains, which is followed by activation of the enzyme invertase (which plays an important role in decreasing sucrose concentration). This activates the FEH enzymes and leads to an increase in the amount of fructose, which in turn induces activation of the 1-FFT enzymes that permit the plant’s stem to flower [1,19,21]. Several authors reported similar results on *A. tequilana* Weber, and found the greatest concentration of fructans in adulthood plants [15,17,19]. Additionally, a reduction in DRS was observed in the older wild plants of all varieties along with an increase in fructan concentration and a greater dispersion. This effect is related to the decreased activity of the FEH enzyme, which permits hydrolysis of the linear and branched fructan chains via the action of the fructosyltransferase enzymes [1,17,19]. Arrizon et al. (2010) [17] reported similar results for *A. tequilana* Weber Blue variety, and a higher increase in the concentration of total carbohydrates at early ages was observed as well as a greater accumulation of free sugars in 2- to 4-year-old plants. Among the adult plants of the wild varieties, *A. tequilana* cenizo variety registered the highest concentrations of total carbohydrates. 

**Figure 1:**
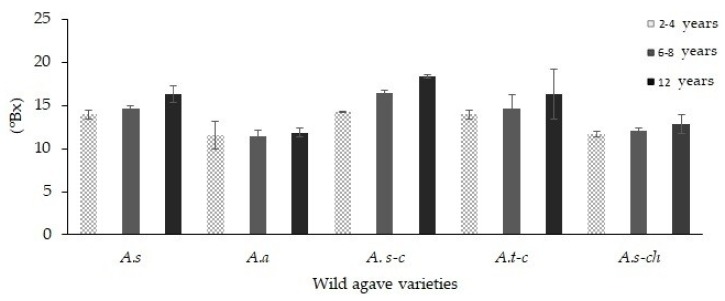
°Brix in the fructan extracts of five wild agave varieties at different ages.

The carbohydrate concentration, as well as the distribution profiles of the different agave samples, are influenced by the species and growth conditions, as mentioned for chicory plants [13]. The total soluble solids (°Bx) showed a statistically significant difference (*p* < 0.05) in the different wild plants analyzed (Figure 1). We found in all cases that °Bx showed no significant difference due to plant age. These results agree with those reported by Mellado-Mojica et al. (2009) [19]. The maximum total soluble solids reported for the evaluated varieties ranged between 11.4 and 18.3 °Bx, which is very close to the data reported for wild agave varieties [22]. These results are also related to different factors, such as the species variety, plant ripeness and harvest season, as well as the climatic conditions in which they grow [13,14,23,24]. The pH was evaluated as a quality parameter and agrees with Norma Oficial Mexicana NOM-002. The pH was 5.95 ± 0.14, and pH values did not show a statistically significant difference (*p* > 0.05) among agave species and plant ages (data not shown). 

### 3.2. Mid-Infrared Spectroscopy (MID-IR) and Principal Component Analysis (PCA) 

**Figure 2:**
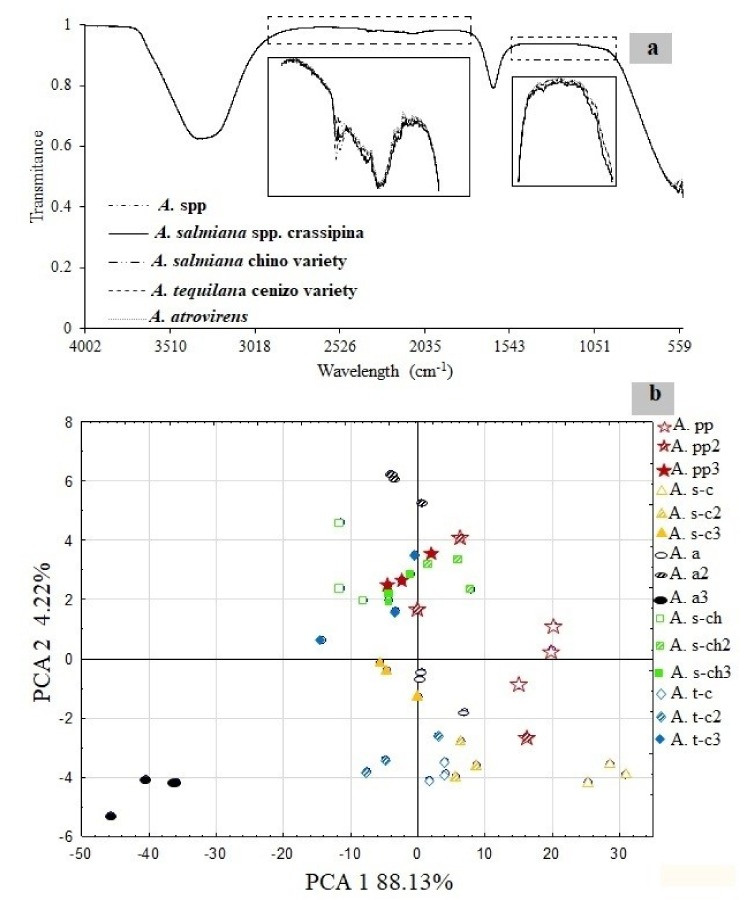
(**a**) Mid-infrared spectroscopy of fructan extracts of five wild agave varieties. (**b**) Principal component analysis of the fructan extracts of five wild agave varieties using only the carbohydrate region (1300–900 cm^−1^).

PCA analysis was carried out using the MID-IR spectra collection (Figure 2a). The agave varieties were classified using only the specific carbohydrate region (1300–900 cm^−1^) instead of the whole spectrum. Figure 2a, shows the spectra of all fructan extracts. Apolinário et al. (2017) [25] reported the presence of hydroxyl groups (−OH) of carbohydrates, with the first band around 3300 cm^−1^. On the other hand, Cui et al. (2014) [26] reported a band around 1591 corresponding to the lignin presence. The presence of other bands, such as those at 1410 and 1100–800 cm^−1^ show the presence of CH_2_-OH bending and stretching of C-C and C-O, respectively [4,25,26,27]. At first look, it seems there are no differences among the five analyzed agave samples. However, PCA showed real differences (Figure 2b). 

Based on the PCA (Figure 2b), it was possible to classify all samples using age and agave variety as criteria. Two specific sections were detected, one possibly highly related to growing agave samples (2–4 years and 6–8 years) with a greater concentration of sugar reported in the positive values of component 1. On the other hand, in the adult plants, negative values predominated component 1 of the graphics, presenting a greater concentration of fructans (Table 1). Component 2 may be related to the information about the differences on the degree of polymerization. A close proximity between two groups of the *A. tequilana* cenizo variety (A. t-c) and *Agave salmiana* spp. crassipina (A. s-c) located in the negative values was observed. It is possible to group *A. salmiana* chino variety (A. s-ch), *Agave atrovirens* (A. a) and *Agave* spp. (A. s) on the positive quadrant (Figure 2b) with an overlap among the samples.

Most samples shared PCA 1 with 88.13%, except for A.s-c and A.a3, and this behavior is not easy to explain since these two samples did not share any characteristics. Finally, a small dispersion between plants replicates can also be seen, mainly those belonging to the same variety (A. spp-2 and A. t-c3). The dispersion observed among the agave samples could have been influenced by the lack of a harvest index to establish the exact agave age.

### 3.3. Distribution Profile of Fructans 

**Figure 3:**
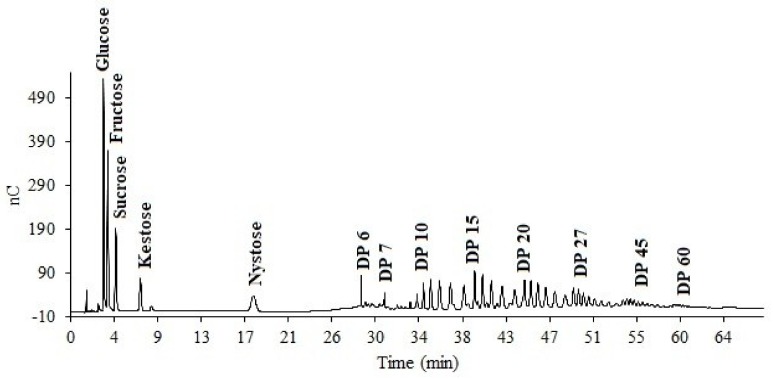
HPAEC-PAD of chicory inulin standard for the apparent Degree of Polymerization determination of Agave fructans.

The specific degree of polymerization among the samples was not possible to be obtain due to the structural complexity presented by the agave fructan structures [28]. In order to determine fructan dispersion and the apparent degree of polymerization (DP_a_) of all the fructan extracts, the elution time of each peak obtained in the profiles of the wild agave fructans was compared to the distribution profile and elution time of a chicory inulin standard (Figure 3).

**Figure 4:**
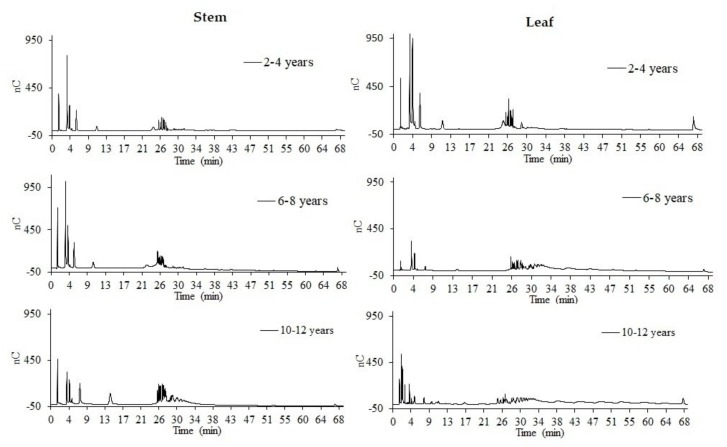
Agave fructan DP_a_ dispersion in agave stem and leaf base in different ages of *A. tequilana* cenizo variety.

The distribution profile for agave fructans changed in regards to carbohydrate distribution as a result of to the plant age for all varieties (figure not shown). *A. tequilana* cenizo variety was considered as a representative example for DP distribution (Figure 4). Sugar-free and low-molecular-weight fructans with a maximum 6 DP_a_ of were predominant in 2- to 4-year-old agaves. On the other hand, fructans reached dispersions up to 15 DP_a_ for 6- to 8-year-old agaves. Finally, at adulthood (10–12 years old), the longest fructan chains were observed after 60 minutes corresponding to a DP_a_ of 70 compared to the inulin standard (Figure 3; Table 1). The degree of polymerization is a function of the fructosyltransferase enzymes, whose activity varies as a function of the plant age. This behavior is related to the energy requirements for growth and protection from extreme conditions where the plant grows [19]. 

Similar results were reported by Arrizon et al. (2010) [17] and Mellado-Mojica et al. (2009) [19], who characterized the species of *A. tequilana* Weber Blue variety at different ages. It was concluded that the carbohydrate profiles changed in comparison to plant age, finding that the youngest plants (2 years old) had the highest concentrations of free sugars and low molecular weight fructans, results that are shown in a quantitate form in Table 2.

Additionally, the distribution profiles of agave fructans from the agave stem and leaf base were determined, with the highest total carbohydrate concentration found in the agave stem. This distribution is found among ages generally (Figure 4). The agave heart presented a lower concentration of simple sugars and a higher concentration of agave fructans, whereas the leaf base showed a higher concentration of simple sugars and fructooligosaccharides (FOSs). The synthesis of oligofructans occurs in the base of agave stems via the activity of the fructosyltransferase enzymes; they accumulate in the vascular tissue for a later transport to the interior of the agave plant stem, where they act as an energy reserve material [20]. Ortiz-Basurto et al. (2008) [29] obtained similar results with the species *Agave mapisaga* from Puebla, Mexico, reporting that fructan concentration was greater in the center of the stem (agave heart) and presented up to 21 g·Kg^−1^, while the base only presented 9.6 g·Kg^−1^. Praznik, Löppert, Cruz, Zangger and Huber (2013) [30] reported 13% fructans and predominantly low DP_a_ for leaves from *A. tequilana* Weber. In complement, Montañez-Soto, Venegas-González, Vivar-Vera and Ramos-Ramírez (2011) [31] reported a concentration of up to 23% in the stem of *A. tequilana*. This confirms a higher fructan concentration in the stems of agave.

**Figure 5:**
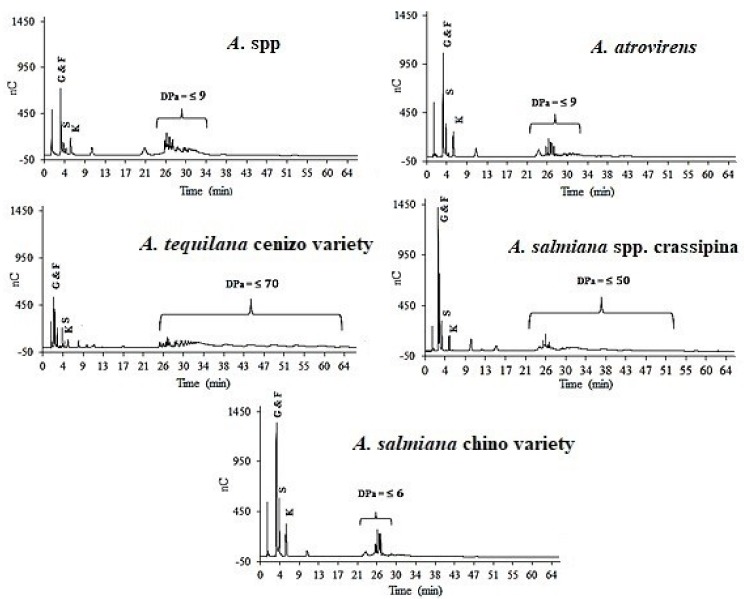
Fructan DP_a_ dispersion of five wild agave varieties in adulthood (G, Glucose; F, Fructose; S, Sucrose; K, Kestose; N, Nystose).

The distribution profiles of the different varieties of wild agave (Figure 5) elucidates that the agave varieties from Veracruz, Mexico (*A.* spp. and *A. atrovirens*) and Guanajuato, Mexico (*A. salmiana*) have a maximum dispersion of 9 DP_a_. Contrary to these results, *A. tequilana* cenizo variety from Jalisco, Mexico (excluded from the DOT) and *A. salmiana* spp. crassipina from Veracruz, Mexico showed distribution profiles of 70 and 50 DP_a_, respectively. These distribution profiles are in accordance with the clusters observed in the PCA of the five agave varieties studied in this work (Figure 2b).

In Table 2, the relative areas of the range of polymerization degree for each agave variety corroborate the behavior of the distribution profile (Figure 4). The DRS diminished according to plant age due to the fructosyltransferase enzyme activity on the polymerization of the fructan chains. Moreover, in the adult stage, a higher concentration of fructans was found compared to young plants.

## 4. Conclusions

In the five wild agave varieties studied in this work, the total concentration of carbohydrates always increased with the age of the plant. The highest concentration of free sugars and lowest degree of polymerization was found in young agaves (2–4 years old) independently of agave variety. The highest concentration of carbohydrates with the greatest dispersion was found in adult plants (10–12 years old) along with a reduction in the concentration of free sugars. The principal component analysis allowed a classification in respect to stretching peaks that differentiate the agave varieties. This last nondestructive technique presents a powerful tool to identify food products produced with different agave materials.

## Figures and Tables

**Table 1:** Sugar content of five wild agave varieties at three different ages.

*Agave* Variety	Age (Years)	Leaf to Leaf Length (cm)	Nomenclature	Direct Reducing Sugars (g·100 g^−1^)	Fructans (g·100 g^−1^)
*A*. spp.	2–4	57.0 ± 11.34 ^a^	A.s1	23.63 ± 0.15 ^a^	76.40 ± 0.15 ^a^
*A*. spp.	6–8	183.0 ± 8.64 ^c^	A.s2	17.33 ± 0.3 ^b^	82.67 ± 0.3 ^b^
*A*. spp.	10–12	255.33 ± 49.10 ^d^	A.s3	14.06 ± 0.12 ^c^	85.90 ± 0.12 ^c^
*A. atrovirens*	2–4	51.66 ± 13.91 ^a^	A.a1	24.21 ± 0.40 ^a^	75.80 ± 0.40 ^a^
*A. atrovirens*	6–8	246.33 ± 55.40 ^d^	A.a2	12.72 ± 0.65 ^c^	87.28 ± 0.65 ^c^
*A. atrovirens*	10–12	322.66 ± 1.24 ^f^	A.a3	13.55 ± 0.23 ^c^	86.45 ± 0.23 ^c^
A. *salmiana* spp. crassipina	2–4	74.0 ± 14.16 ^ab^	A.s-c1	22.70 ± 1.06 ^a^	77.30 ± 1.06 ^a^
A. *salmiana* spp. crassipina	6–8	264.0 ± 18.49 ^de^	A.s-c2	11.92 ± 0.92 ^c^	88.08 ± 0.92 ^c^
A. *salmiana* spp. crassipina	10–12	308.0 ± 14.89 ^ef^	A.s-c3	13.08 ± 1.34 ^c^	86.90 ± 1.34 ^c^
A. *tequilana* cenizo variety	2–4	56.83 ± 4.36 ^a^	A.t-c1	21.30 ± 1.16 ^a^	78.30 ± 1.16 ^a^
A. *tequilana* cenizo variety	6–8	97.5 ± 24.60 ^ab^	A.t-c2	18.55 ± 0.45 ^b^	81.45 ± 0.45 ^b^
A. *tequilana* cenizo variety	10–12	85.66 ± 7.76 ^ab^	A.t-c3	10.38 ± 0.87 ^c^	89.70 ± 0.87 ^c^
A. *salmiana* chino variety	2–4	104.33 ± 14.61 ^b^	A.s-ch1	26.40 ± 1.07 ^a^	73.60 ± 1.07 ^a^
A. *salmiana* chino variety	6–8	163.0 ± 14.56 ^c^	A.s-ch2	18.03 ± 0.84 ^b^	81.97 ± 0.84 ^b^
A. *salmiana* chino variety	10–12	187.83 ± 21.83 ^c^	A.s-ch3	13.08 ± 0.77 ^c^	86.90 ± 0.77 ^c^

Different letters indicate significant statistical differences between lines. Statistical analysis was performed by column, leaf to leaf (*p* = 0.023979), Direct Reducing Sugars (*p* = 0.005049).

**Table 2:** Relative area value of the degree of polymerization of agave fructans.

Variety	Age (years)	DP_a_ 1–2	DP_a_ 3–9	DP_a_ 10–39	DP_a_ > 40
*A*. spp.	2–4	48.69 ± 7.91	30.21 ± 8.66	10.13 ± 4.61	---
*A*. spp.	6–8	41.47 ± 4.76	53.73 ± 22.35	---	---
*A*. spp.	10–12	28.21 ± 14.47	40.06 ± 6.86	6.46 ± 3.71	---
*A. atrovirens*	2–4	38.11 ± 5.03	35.65 ± 4.57	10.82 ± 5.32	4.53 ± 1.19
*A. atrovirens*	6–8	34.05 ± 9.83	41.79 ± 6.2	1.41 ± 0.09	---
*A. atrovirens*	10–12	27.99 ± 8.09	49.51 ± 14.19	2.08 ± 1.42	---
A. *salmiana* spp. crassipina	2–4	22.16 ± 0.60	51.84 ± 17.53	---	---
A. *salmiana* spp. crassipina	6–8	44.53 ± 10.65	36.80 ± 8.25	1.43 ± 0.17	---
A. *salmiana* spp. crassipina	10–12	25.05 ± 12.62	32.66 ± 9.51	4.3 ± 1.37	---
A. *tequilana* cenizo variety	2–4	41.11 ± 1.56	49.32 ± 5.79	2.16 ± 1.08	---
A. *tequilana* cenizo variety	6–8	21.06 ± 1.50	60.77 ± 4.59	10.73 ± 5.96	4.20 ± 1.19
A. *tequilana* cenizo variety	10–12	14.21 ± 1.45	28.04 ± 7.93	43.10 ± 1.74	14.53 ± 2.12
A. *salmiana* chino variety	2–4	34.55 ± 12.22	39.56 ± 8.53	4.37 ± 1.15	---
A. *salmiana* chino variety	6–8	30.19 ± 6.23	50.76 ± 4.93	2.75 ± 1.53	---
A. *salmiana* chino variety	10–12	22.76 ± 13.66	56.07 ± 14.61	3.46 ± 0.57	---

## Abbreviations

DP_a_	Apparent degree of polymerization
DRS	Direct reducing sugar
FEH	Fructan 1-exohydrolase
1-FFT	Fructan:fructan 1-fructosyltransferase
6-SFT	Sucrose:fructan 6-fructosyltransferase
